# Production and New Extraction Method of Polyketide Red Pigments Produced by Ascomycetous Fungi from Terrestrial and Marine Habitats

**DOI:** 10.3390/jof3030034

**Published:** 2017-06-28

**Authors:** Juliana Lebeau, Mekala Venkatachalam, Mireille Fouillaud, Thomas Petit, Francesco Vinale, Laurent Dufossé, Yanis Caro

**Affiliations:** 1 Laboratoire de Chimie des Substances Naturelles et des Sciences des Aliments (LCSNSA), Université de la Réunion, F-97490 Sainte-Clotilde, Ile de la Réunion, France; juliana.lebeau@gmail.com (J.L.); mekalavenkat@gmail.com (M.V.); mireille.fouillaud@univ-reunion.fr (M.F.); laurent.dufosse@univ-reunion.fr (L.D.); 2 UMR QualiSud, Université de la Réunion, IUT, F-97410 Saint-Pierre, Ile de la Réunion, France; thomas.petit@univ-reunion.fr; 3 Istituto per la Protezione Sostenibile delle Piante (IPSP-CNR) and Dipartimento di Agraria, Università degli Studi di Napoli Federico II, 80055 Portici, NA, Italy; francesco.vinale@ipsp.cnr.it

**Keywords:** red pigment, fungal pigment, *Talaromyces*, *Penicillium*, marine fungi, ascomycetous, *N*-threoninerubropunctamine

## Abstract

The use of ascomycetous fungi as pigment producers opens the way to an alternative to synthetic dyes, especially in the red-dye industries, which have very few natural pigment alternatives. The present paper aimed to bio-prospect and screen out 15 selected ascomycetous fungal strains, originating from terrestrial and marine habitats belonging to seven different genera (*Penicillium*, *Talaromyces*, *Fusarium*, *Aspergillus*, *Trichoderma*, *Dreschlera*, and *Paecilomyces*). We identified four strains, *Penicillium purpurogenum rubisclerotium*, *Fusarium oxysporum*, marine strains identified as *Talaromyces* spp., and *Trichoderma atroviride*, as potential red pigment producers. The extraction of the pigments is a crucial step, whereby the qualitative and quantitative compositions of each fungal extract need to be respected for reliable identification, as well as preserving bioactivity. Furthermore, there is a growing demand for more sustainable and cost-effective extraction methods. Therefore, a pressurized liquid extraction technique was carried out in this study, allowing a greener and faster extraction step of the pigments, while preserving their chemical structures and bioactivities in comparison to conventional extraction processes. The protocol was illustrated with the production of pigment extracts from *P. purpurogenum rubisclerotium* and *Talaromyces* spp. Extracts were analyzed by high-performance liquid-chromatography combined with photodiode array-detection (HPLC-DAD) and high-resolution mass spectrometry (UHPLC-HRMS). The more promising strain was the isolate *Talaromyces* spp. of marine origin. The main polyketide pigment produced by this strain has been characterized as *N*-threoninerubropunctamine, a non-toxic red *Monascus*-like azaphilone pigment.

## 1. Introduction

Natural colorants are widely used in the world in many industries such as food, cosmetics, pharmaceuticals, and textiles. The majority of authorized natural food colorants in the market are of either a plant or vegetable origin, and have numerous drawbacks such as instability against light, heat, or adverse pH, and a low water solubility [1]. The dye industry is currently suffering from the cost increase of feedstock associated with the higher demands of eco-friendly pigments for replacing synthetic dyes (like azo dyes). This is even more the case in the red-dye industries, which have no, or very few, natural red pigment alternatives for food processes. For instance, there is a strong need for red colours other than plant-originated anthocyanins, which cannot be used over the whole pH range. As of now, red pigments used in foods are mainly from insects (carmine). Carmine (or carminic acid, cochineal extract) is produced in Peru, Bolivia, Mexico, Chile, and Spain (Canary islands) from the dried bodies of female cochineal insects (*Dactylopius coccus*), primarily grown on *Opuntia* cacti [2]. Carmine is considered as one of the most stable natural food red colorants in terms of light and heat. Between 2004 and 2009, a 76% increase in new European food product launches listing carmine as an ingredient was observed, an increase also linked to the consequences of the “Southampton six” study, which promoted a warning for child hyperactivity related to the occurrence of six artificial colorants in food, including three sulphonated mono azo red dyes (E122 carmoisine/azorubine, E124 Ponceau 4R, and E129 Allura Red AC). However, carmine holds ethical issues for some social groups, and another drawback of carmine products is that from a stable level of 15 USD per kg, it surged in 2010–2011 up to 120 USD per kg and decreased again to 15 USD per kg. As a conclusion, “Dr Jekyll’s” (positive) aspect of carmine is its excellent stability in food formulations, whereas the “Mr Hyde” (negative) ones are: (i) it cannot be used by vegans-vegetarians-kosher-halal, (ii) its price versatility, and (iii) allergenicity in some cases [3]. The world’s largest food color company, Chr. Hansen, which sources one third of global carmine production, decided in 2011 to explore whether it would be commercially viable to produce carmine with a controlled fermentation process (proof of concept test).

Thus, there is an increasing interest from the academic world and industrial sector about the readily available natural sources of red pigments. Among non-conventional sources, ascomycetous fungi are known to produce an extraordinary range of red polyketide pigments that are often more stable and soluble than plant pigments [4,5,6]. So, fungal red polyketides, such as azaphilone, naphtoquinone, and hydroxyanthraquinone red compounds, are most promising in this respect, even if unusual microbial red carotenoids should be investigated. The development of such a fungal-based pigments industry and its sustainability rely on the selection of adequate strains regarding the three following parameters: (i) profitable yields, (ii) pigment purity and stability, (iii) and the total absence of toxic compounds in the fungal pigment extract. Furthermore, fungal pigments are of interest due to the broad spectrum of their biological activities and their potential applications in designing new pharmaceutical products [7].

Nowadays, some fermentative natural colorants from filamentous fungi like *Blakeslea trispora*, *Ashbya gossypii*, *Penicillium oxalicum*, and *Monascus* sp., are available for replacing the yellow, orange, and red synthetic dyes [6,7,8,9]. Over the past five years, very few reports have been published on the *Monascus*-like azaphilone red pigments produced by non-mycotoxigenic strains of *Talaromyces* species [6,7,8,9]. In the literature, this biosynthetic potential has been linked to species such as *Talaromyces purpurogenus*, *T. albobiverticillius*, *T. marneffei*, and *T. minioluteus*, often known under their previous *Penicillium* names. For example, in 2012, a European patent was granted for a submerged cultivation method for some of the non-mycotoxigenic strains of *Talaromyces* sp., whereby the concentration of pigments was significantly enhanced, with the polyketide azaphilone purple pigment PP-V [(10*Z*)-12-carboxyl-monascorubramine] being the major compound [10,11,12]. *N*-glutarylmonascorubramine and *N*-glutarylrubropunctamine were the water-soluble *Monascus*-like polyketide azaphilone red pigments discovered in the extracellular pigment extract obtained from the liquid medium of *Penicillium purpurogenum* [13]. Recently, Frisvad et al. [14] concluded that the isolate of *T. atroroseus* sp. nov., which produces *Monascus*-like azaphilone red pigments and mitorubrins, without being accompanied by mycotoxin synthesis, can be used industrially for red pigment production (patent application EP2262862 B1 [12]). However, they indicated that isolates identified as *T. purpurogenus* may not be recommended for the industrial production of red pigments due to their potential coproduction of mycotoxins, such as rubratoxin A and B, and luteoskyrin, in addition to potential toxic extrolites, such as spiculisporic acid and rugulovasine A and B.

Few reports have been published on the following polyketide naphthoquinone red pigments produced by *Fusarium* species: aurofusarin in *Fusarium graminearum* [15] and bikaverin and its minor coproduct nor-bikaverin in *Fusarium fujikuroi* [6,16]. Along similar lines, some species of the genus *Aspergillus* were found to produce known polyketide hydroxyantraquinone red pigments, such as erythroglaucin, catenarin, and rubrocristin [2,6,17]. Some strains of *Trichoderma* such as *T. aureoviride*, *T. harzianum*, and *T. polysporum* are found to produce the hydroxyanthraquinone orange-red pigment chrysophanol [6]. The hydroxyanthraquinone red pigments catenarin and erythroglaucin have also been isolated from cultures of strains among *Drechslera* species and from a culture of *Curvularia lunata* [6].

The present paper aimed to bio-prospect and screen out 15 selected ascomycetous fungal strains, belonging to seven different genera (*Penicillium*, *Talaromyces*, *Fusarium*, *Aspergillus*, *Trichoderma*, *Dreschlera*, and *Paecilomyces*) originating from terrestrial and marine habitats. Recent literature abundantly reports the interest in marine microorganisms with respect to the production of new molecules and, among them, new pigments [7]. The biotechnological properties of the 15 strains for the production of extracellular water-soluble pigments and intracellular polyketide pigments were investigated in submerged cultures.

The choice of extraction protocol is crucial, as the extraction solvents and conditions can drastically influence the final composition, quality, and efficiency of the process. Indeed, extended extraction times, and the exposure to organic solvents and a higher temperature, can result in a tremendous loss of bioactive substances due to hydrolysis, oxygen- and light-oxidation, as well as ionization. In order to preserve as much as possible of the qualitative and quantitative compositions of the pigmented molecules, the use of a recent extraction method, known as Pressurized Liquid Extraction, was investigated, resulting in the development of a six-stage pressurized liquid extraction protocol (PLE) for advanced mycelial pigment extraction. PLE has been mostly applied on environmental samples (recovery) [18], as well as food and biological samples [19], as an analytical method. Only a few applications have been reported on the extraction of bioactive phyto-compounds. Thus, to our knowledge, such methods, which use less and non-toxic solvents, have not been used on fungal matrixes thus far.

## 2. Materials and Methods

### 2.1. Fungal Strains

Eleven fungal strains used in this study originating from terrestrial environments were bought from the fungal culture collection of the Museum d’Histoire Naturelle de Paris (Paris, France): *Penicillium purpurogenum* LCP4890, *P. purpurogenum rubisclerotium* LCP4464, *P. erythromellis* LCP3684, *P. oxalicum* LCP4158, *Fusarium oxysporum* LCP531, *Aspergillus repens* LCP5511, *Paecilomyces farinosus* LCP3391, *Trichoderma harzianum* LCP3404, *T. polysporum* LCP3531, and *Dreschlera cynodontis* LCP2226. *Trichoderma harzianum* strain T22 is a commercial biological control strain. The four fungal isolates of marine origin investigated in this study and identified as *Talaromyces* spp. (code: 305_70), *Talaromyces verruculosus* (code: PA9), *Trichoderma atroviride* (code: 305_55), and *Aspergillus sydowii* (code: B34) were isolated by Mireille Fouillaud from samples collected in the back reef-flat and on the external slope of the coral reef on the west coast of La Reunion island. The fungal collection was stored at −80 °C at the LCSNSA laboratory (Reunion island).

### 2.2. Fermentation and Biomass Production

For inoculum preparation, 0.15 g of conidia and mycelium mixture was sampled from a seven days-old preculture on a potato dextrose agar (PDA) plate, and transferred into a microcentrifuge tube containing 1 mL of nutrient broth supplemented with 0.05 g·L^−1^ of Tween^®^ 80 (Sigma-Aldrich Co, Saint Louis, MO, USA). The mycelium was crushed and the suspension was used to inoculate 250-mL flasks containing 100 mL of liquid media: (i) potato dextrose broth medium (PDB: composed of 4 g·L^−1^ potato infusion solids and 20 g·L^−1^ glucose; Sigma-Aldrich); (ii) defined minimal dextrose broth medium (DMD: composed of 1 g·L^−1^ ammonium sulfate, 30 g·L^−1^ glucose, 0.5 g·L^−1^ MgSO_4_, 1.4 g·L^−1^ K_2_HPO_4_, 0.6 g·L^−1^ KH_2_PO_4_, 0.8 mg·L^−1^ ZnSO_4_, 0.8 mg·L^−1^ FeSO_4_, 0.8 mg·L^−1^ CuSO_4_, 0.8 mg·L^−1^ NaH_2_PO_4_ and 0.4 mg·L^−1^ MnSO_4_; Fisher Scientific UK Limited, Loughborough, Leicestershire LE, UK) based on Velmurugan et al. [20]; and (iii) yeast casamino dextrose broth (YCD: composed of 1 g·L^−1^ yeast extract (Becton, Dickinson and Co., Sparks, MD, USA), 5 g·L^−1^ casamino acids (BD Bacto), 20 g·L^−1^ glucose, 5 g·L^−1^ sodium chloride and 1 g·L^−1^ KH_2_PO_4_ (Fisher Scientific UK Limited, Loughborough, Leicestershire LE, UK) based on Guyomarc’h et al. [21]. The pH was adjusted to 6.0. Flasks were incubated at 26 °C and agitated at 150 rpm. For cultures performed in total darkness, flasks were wrapped in aluminum foil. After seven days of fermentation, all the contents of each flask were collected and centrifuged at 10,000 rpm for 10 min; the resulting supernatant was filtered through a Whatman filter paper (GF/C) at a reduced pressure using a Büchner funnel to obtain the culture filtrate. The mycelial biomass was washed with deionized water. After freezing at −84 °C in an ultra-low-temperature freezer (Sanyo, Guangzhou, China) for at least 2 h, the samples were quickly transferred to a LABCONCO FreeZone 2.5 lyophilizer (LABCONCO, Kansas City, MO, USA) and lyophilized for 24 h. During freezing, the condenser temperature and vacuum pressure were maintained at −47 °C and 200 mbar, respectively. Then, dried cells were weighed to estimate the biomass. All experiments were conducted in duplicate.

### 2.3. Quantitative Colorimetric Analysis of Extracellular Extracts

The colorimetric characterization of extracellular extracts was assessed from the pigmented culture filtrate after seven days of cultivation. Measurements were performed in the CIE L*a*b* (L*a*b* colorimetric system of the Commission Internationale de l’Eclairage) using a spectrocolorimeter CM 3500 with the SpectraMagic^TM^ software v1.9 (Konica Minolta, Mahwah, NJ, USA). The so-called CIELab colorimetric system is based on the fact that light reflected from any colored surface can be visually matched by an additive mixture of the three primary colors: red, green, and blue [22,23]. To characterize a color in the CIE L*a*b* color system, three colorimetric coordinates are obtained from the spectrocolorimeter. L* defines the lightness (ranges from 0% to 100%, dark to light), a* value indicates the red/green value (from −60 to +60, green to red), and b* value denotes the blue/yellow value (from −60 to +60, blue to yellow). The attributes of color, C* and h°, describe the chroma (vividness or dullness) and the hue angle (or tone) of the color, respectively. The value of chroma C* is 0 at the center and increases according to the distance from the center. The hue angle h° is defined as starting at the +a* axis and is expressed in degrees: 0° would be +a* (red), 90° would be +b* (yellow), 180° would be −a* (green), and 270° would be −b* (blue). Hue values correspond to the angle of the a*/b* coordinates of the points. The “Y red chroma” value used in this study to link the positive a*-value (red color) and the others coordinates and attributes of the color of the pigment extract (such as b*, C* and h* values) was calculated as follow:

“Y red chroma” value = f (a* value) = b* value × C* chroma value × 1/h*(1)

### 2.4. UV-Visible Spectrophotometry and Extracellular Polyketide Metabolites Quantification

The culture filtrate was diluted in deionized water with a dilution factor (d) ranging from four to 10 with respect to the concentration of extrolites in the filtrate. The solution was used to investigate the sample absorption profile in the wavelength range of 200–800 nm. Then, the concentrations of polyketide metabolites in the culture filtrate were determined through the absorption values read at 276 nm using a UV-visible spectrophotometer (UV-1800, Shimadzu Corporation, Tokyo, Japan). Polyketide red carmine aqueous solutions were used as standards, and their maximum absorption values experimentally measured at 276 nm were taken as the reference for polyketide concentration determination (Figure S1). Carmine (hydrosoluble carmine in powder, kindly provided by Pronex, Lima, Peru) used as the standard was dissolved in pure water. The diluted uninoculated liquid broth (that is PDB, DMD, or YCD broth) was used as a blank before any absorbance measurements were taken. The coefficient of proportionality (ε), which links the absorbance at 276 nm with the extracellular polyketide metabolites concentration, was obtained by linear regression (Figure S1). The concentration of extracellular polyketide metabolites in culture filtrate was expressed as milli-equivalents of polyketide carmine per liter of liquid broth.

### 2.5. New Extraction Method of Intracellular Polyketide Pigments

The extraction of intracellular polyketide pigments from the mycelial biomass was performed using a new pressurized liquid extraction (PLE) process. The weighed sample (lyophilized biomass) was transferred to a 10-mL stainless steel extraction cell equipped with two cellulose filters on the bottom and containing glass balls (diam. 0.25–0.50 mm). PLE extraction was performed on a Dionex ASE system (ASE^TM^ 350 apparatus, Dionex, Germering, Germany). The ASE conditions were: temperature: 90 °C, pressure: 1500 psi, heating time: 5 min, static time: 18 min, flush: 100%, and purge: 5 min. The lyophilized biomass was subjected to a six-stage liquid solvent extraction under pressure as an attempt to entirely extract the intracellular pigments from the mycelium. The following six-stage PLE sequence was performed: purified water was used as the first extraction solvent, followed by 50% methanol, then 50% ethanol, >99.9% methanol, and MeOH:EtOH (1/1, *v*/*v*), and then, mycelium was depleted with >99.9% ethanol as the extraction solvent (Figure S2). The sequence of solvents was set to show a decreasing polarity profile. Solvents (methanol and ethanol, 99.9%-HPLC quality) were obtained from Carlo Erba (Val de Reuil, France). Purified water was obtained from a Milli-Q system (EMD Millipore Co., Billerica, MA, USA). A part of each color extract was used for chromatographic analysis and the rest was used for absorbance analysis.

### 2.6. Absorbance and HPLC-DAD Analyses

Each intracellular extract obtained in a collection bottle at the end of the six-stage pressurized liquid solvent extraction sequence was filtered onto a 0.2-µm poresized hydrophylic Millex-GV membrane (Millipore, Carrigtwohill, Ireland) and stored at −20 °C in amber glass vials (2 mL) with Teflon-lined caps, until further analysis. The total polyketide secondary metabolite content extracted from the mycelial biomass was first analyzed by measuring the absorbance of each extract by spectral analysis at 276 nm. The results obtained are expressed in terms of meqv. of carmine per g dry cell mass, a value proportional to the polyketide metabolite concentration extracted from the mycelia. Then, reverse phase high-resolution liquid-chromatography combined with photodiode array-detection (HPLC-DAD) analysis was performed on each extract (25 µL injection) on a Dionex HPLC-DAD system (Ultimate 3000 apparatus, Dionex, Germering, Germany) and using a Hypersil Gold^TM^ column (150 mm × 2.1 mm i.d., 5 μm; Thermo Scientific Inc., Waltham, MA, USA) maintained at 30 °C. The HPLC-DAD system was operated using a (A) purified water-(B) acetonitrile-(C) aqueous formic acid 1% (*v*/*v*) solution gradient system starting from a ratio of 45%(A)–45%(B)–10%(C) for 8 min and increasing to 95%(B)–5%(C) in 55 min, at which point it was maintained for 20 min. The flow rate was 0.4 mL·min^−1^. Monitoring, data recording, and processing were led with the Chromeleon v.6.80 software (Dionex). Solvents (acetonitrile and methanol, 99.9%-HPLC quality) and formic acid (purity 99%) were obtained from Carlo Erba (Val de Reuil, France).

### 2.7. UHPLC-HRMS Analyses

The intracellular extracts were analyzed by ultra high performance liquid chromatography high-resolution mass spectrometry (UHPLC-HRMS) according to Klitgaard et al. [24]. Liquid chromatography was performed on an Agilent 1290 Infinity LC system with a DAD detector, coupled to an Agilent 6550 iFunnel Q-TOF with an electrospray ionization source (Agilent Technologies, Santa Clara, CA, USA). The separation was performed on a 2.1 mm × 250 mm, 2.7 μm Poroshell 120 Phenyl-Hexyl column (Agilent) at 60 °C with a water-acetonitrile gradient (both buffered with 20 mM formic acid) going from 10 % (*v*/*v*) to 100 % acetonitrile in 15 min, followed by 3 min with 100 % acetonitrile. The flow rate was kept constant at 0.35 mL/min throughout the run. The injection volume, depending on the sample concentration, typically varied between 0.1 and 1 μL. Mass spectra were recorded as centroid data for *m*/*z* 85–1700 in MS mode and *m*/*z* 30–1700 in MS/MS mode, with an acquisition rate of 10 spectra/s. The lock mass solution in 95% acetonitrile was infused in the second sprayer using an extra LC pump at a flow of 10–50 μL/min, and the solution contained 1 μM tributyle amine (Sigma-Aldrich), 10 μM Hexakis(2,2,3,3-tetrafluoropropoxy) phosphazene (Apollo Scientific Ltd., Cheshire, UK), and 1 μM trifluoroacetic acid (Sigma-Aldrich) as lock masses.

## 3. Results

### 3.1. Biomass and Polyketide Extrolites Production Capacities under Various Growth Conditions

**Figure 1:**
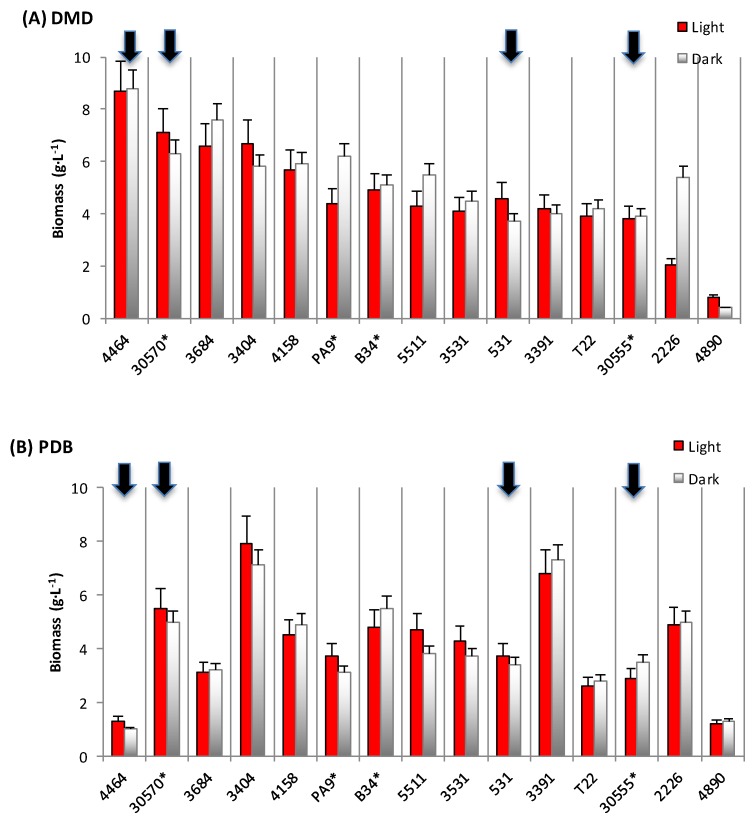

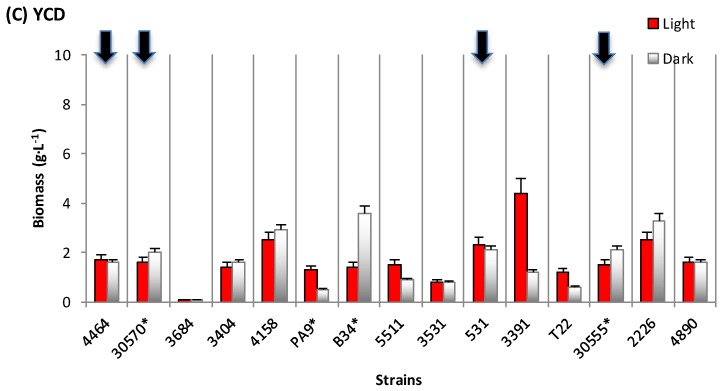
Fungal biomass production (mean in g·L^−1^ ± s.d.) in a submerged culture of the ascomycetous fungi. (**A**) Biomass production obtained in DMD (Defined Minimal Dextrose broth) submerged culture; (**B**) in PDB (Potato Dextrose Broth) submerged culture; (**C**) in YCD (Yeast Casamino Dextrose broth) submerged culture; Culture conditions: under illumination (∎: red) and in the dark (∎: grey); s.d.: standard deviation; Strain identification: *4464 =* ❶ *Penicillium purpurogenum rubisclerotium; 30570 =* ❷ *Talaromyces* spp. (marine isolate)*; *531 =* ❸ *Fusarium oxysporum; 30555 =* ❹ Trichoderma atroviride*; 4890= Penicillium purpurogenum; 2226 = Dreschlera cynodontis; 3684 = Penicillium erythromellis; *T22 = Trichoderma harzianum*; *4158 = Penicillium oxalicum*; *5511= Aspergillus repens*; PA9= Talaromyces verruculosus*; *3404= Trichoderma harzianum*; 3391= Paecylomyces farinosus; B34 = Aspergillus sydowii*; *3531 = Trichoderma polysporum*; (* strains collected from marine biotopes of La Reunion island’s reef flat).

Our results indicated that the 15 fungal strains investigated in the present study could be divided into two main color categories. The reddish polyketide pigment producers include the four following strains, ranked from the darker to the paler red-pigment producers: *P. purpurogenum rubisclerotium*, the local marine isolate identified as *Talaromyces* spp., followed by the strain *F. oxysporum*, and the marine isolate *Trichoderma atroviride*. The yellowish pigment producers encompass the following strains: *P. erythromellis*, *P. oxalicum*, *Talaromyces verruculosus*, *Trichoderma harzianum*, *Paecilomyces farinosus*, *Aspergillus repens*, and *A. sydowii* (Figure S3). The dried biomass concentration produced by the 15 ascomycetous strains is reported in Figure 1. Three different culture broths were investigated: (i) defined minimal dextrose medium (DMD), (ii) potato dextrose broth medium (PDB), and (iii) yeast casamino dextrose broth (YCD).

Our results indicated that the DMD medium (Figure 1A) is the more favorable liquid broth for biomass production. Six fungal strains producing more than 5.5 g·L^−1^ of dry biomass were observed: *P. purpurogenum rubisclerotium*, i.e., the first reddish pigment producer described above, which produced 8.4 and 8.5 g·L^−1^ in the light and in the dark culture, respectively; followed by the marine isolate *Talaromyces* spp. (the second reddish pigments producer: 7.1 and 6.2 g·L^−1^ of dry biomass); and four other strains, i.e., *P. erythromellis* (6.5 and 7.5 g·L^−1^), *Trichoderma harzianum* (6.7 and 5.8 g·L^−1^), *P. oxalicum* LCP4158 (5.7 and 5.9 g·L^−1^), and *T. verruculosus* (4.4 and 6.1 g·L^−1^). The two other reddish pigment producers, i.e., the strain *F. oxysporum* and the marine isolate *T. atroviride*, produced less than 4.5 g·L^−1^ of biomass in this DMD medium. In PDB medium (Figure 1B), considering the four fungal strains identified above as potential reddish pigment producers, only the marine strain *Talaromyces* spp. produced more than 5.5 g·L^−1^ of dry biomass. Surprisingly, whereas the strain *P. purpurogenum rubisclerotium* showed the highest biomass productions in the DMD medium, it was the less productive strain in PDB medium (1.3 and 1.0 g dry biomass·L^−1^ in the light and in the dark culture, respectively). In YCD medium (Figure 1C), the biomass contents remained relatively lower (<2 g·L^−1^) for most of the cultured fungal strains.

**Figure 2:**
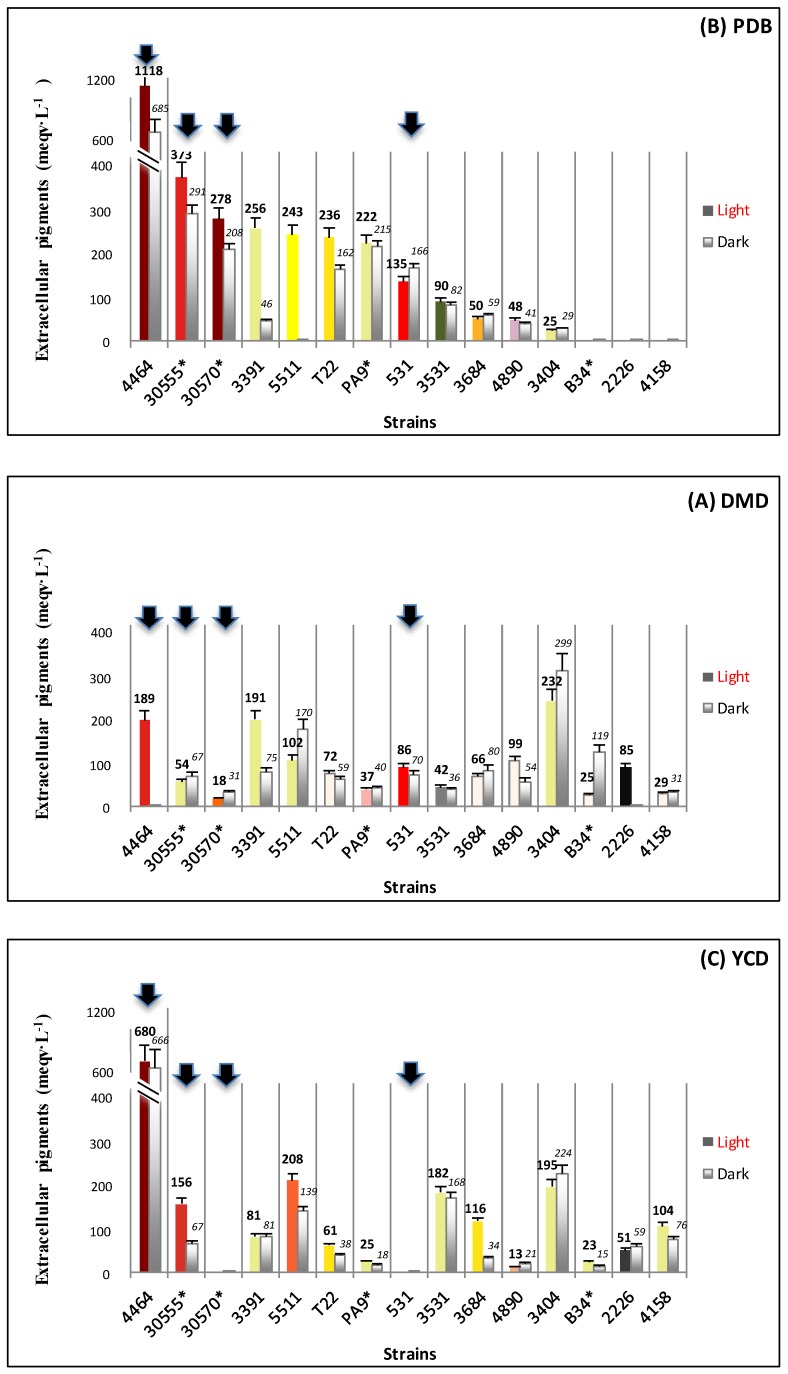
Volumetric production of extracellular pigments (mean in meqv. carmine pigment per liter ± s.d.) by the ascomycetous fungi in submerged culture. (**A**) Volumic production of extracellular pigments obtained in DMD (Defined Minimal Dextrose broth) submerged culture; (**B**) in PDB (Potato Dextrose Broth) submerged culture; (**C**) in YCD (Yeast Casamino Dextrose broth) submerged culture; Culture conditions: in the dark (grey) and under illumination (in color; color in figure for each strain indicates the shade of the fungal culture filtrate after 7 days of fermentation); s.d.: standard deviation; ❶ *Penicillium purpurogenum rubisclerotium* (4464); ❷ *Talaromyces spp.* (marine isolate 30570); ❸ *Fusarium oxysporum* (531); ❹ *Trichoderma atroviride* (marine isolate 30555). For other strain identification (numbers in horizontal axis), see Figure 1. The yield of extracellular pigments was estimated according to a calibration curve of standard carmine solution by measuring the absorbance at 276 nm (λ_max_ of carmine) of the colored culture filtrate (after blank subtraction).

Figure 2 presents an estimation of the volumetric productions of polyketide extrolites secreted by each strain in the culture filtrate. The concentrations were expressed as milli-equivalents of carmine per liter of liquid broth. The color of the “light” histograms of Figure 2 indicates the shade of the culture filtrate after seven days of fermentation: reddish and yellowish shades were mainly observed, but an orange shade (obtained for *A. repens* cultivated in YCD under light conditions), pink shade (*T. verruculosus* grown in DMD in darkness, or *P. purpurogenum* grown in PDB and YCD), brown shade (*Dreschlera cynodontis* grown in DMD and YCD broths), and green shade (*Trichoderma polysporum* cultivated in PDB and DMD broths) were also noticed (S3). The lowest extracellular productivities (<200 meqv g·L^−1^) were observed on “minimal” DMD medium for all strains investigated in this study (Figure 2A). Thus, the presence of a simple source of carbon (30 g·L^−1^ of glucose) and simple source of nitrogen (ammonium sulfate) in this DMD medium favored the production of mycelial biomass to the detriment of the production and excretion of extracellular diffusible polyketides. In contrast, the best polyketide extrolite productions were obtained in PDB liquid culture medium, reaching an average extracellular volumetric production ranging from 135 to 1118 meqv·L^−1^ for eight strains out of the 15 (Figure 2B). For example, the highest extrolite production was obtained in culture filtrate of the strain *P. purpurogenum rubisclerotium* grown in PDB liquid broth (1118 and 685 meqv·L^−1^ in the light and dark culture, respectively), while its biomass concentration was very low in this medium (only 1.0–1.3 g·L^−1^ of dry biomass). The marine isolate *Talaromyces* spp. presents a different behavior towards the medium nutrient composition. It simultaneously showed a relatively high mycelial biomass content (above 5.5 g·L^−1^ of dry biomass) in PDB and a strong content in extracellular polyketide-derived metabolites (278 and 266 meqv·L^−1^ in the light and dark culture, respectively). The two other strains belonging to the reddish pigment producers, i.e., *F. oxysporum* and *T. atroviride* (marine isolate), presented extracellular polyketide productivities of 166 and 373 meqv·L^−1^, respectively, but also low contents in the mycelial biomass in submerged cultures in PDB (< 3.5 g·L^−1^ of dry biomass). The YCD medium that also contained a complex source of nitrogen (f.i. amino acids and proteins from yeast extract) had an intermediate position regarding extracellular polyketide extrolite production, exhibiting very heterogeneous productivities (Figure 2C).

**Figure 3:**
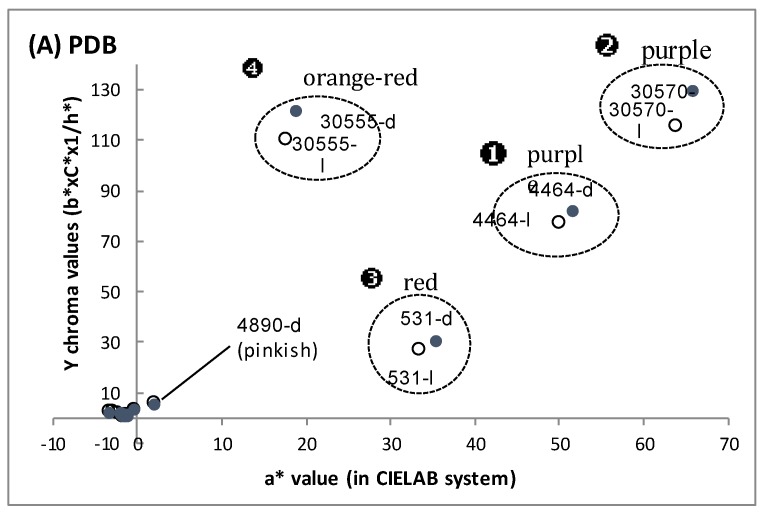

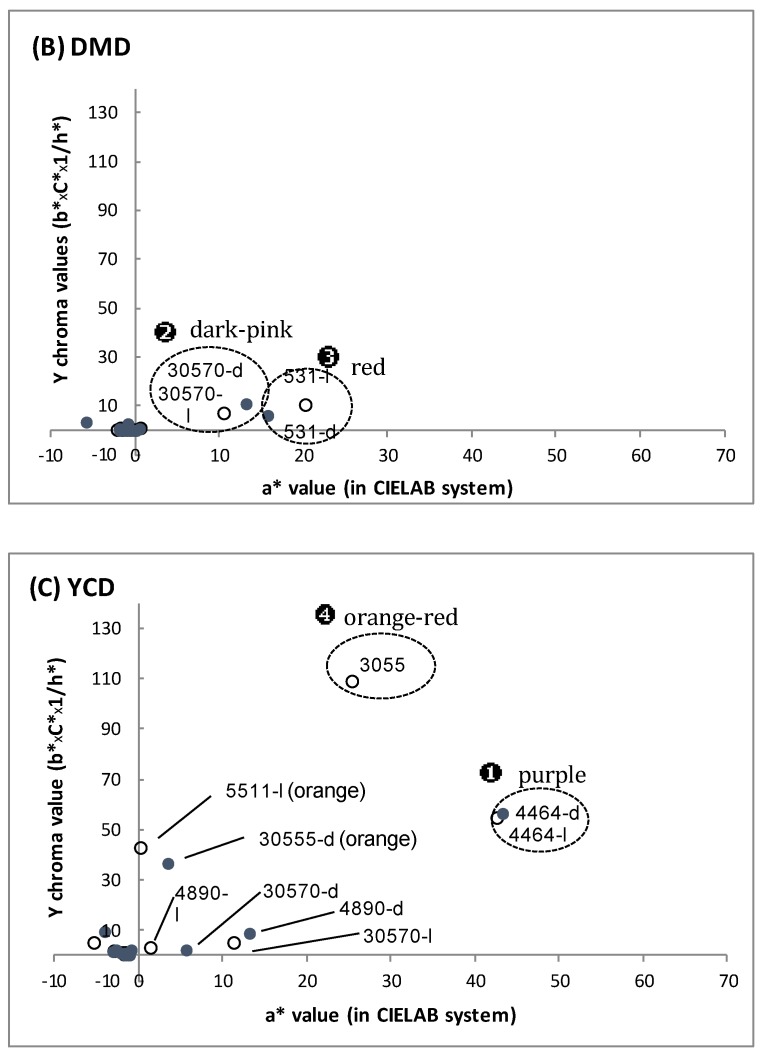
Color coordinates projected in the CIE L*a*b* colorimetric system of the culture filtrates after seven days of cultivation of the ascomycetous fungi. (**A**) Color coordinates of extracellular pigment extracts obtained in PDB (Potato Dextrose Broth) submerged culture; (**B**) in DMD (Defined Minimal Dextrose broth) submerged culture; (**C**) in YCD (Yeast Casamino Dextrose broth) submerged culture; Culture conditions: under illumination (-l), and darkness (-d). Strain identification: ❶ *Penicillium purpurogenum rubisclerotium* (4464); ❷ *Talaromyces spp.* (marine isolate 30570); ❸ *Fusarium oxysporum* (531); ❹ *Trichoderma atroviride* (marine isolate 30555); *Penicillium purpurogenum* (4890).

The culture filtrates were then analyzed by quantitative colorimetric analysis in the CIE L*a*b* colorimetric system. It is worth noticing that for colored culture filtrates, the greater the positive red a*-value is, the greater the positive b*-value, the greater the positive C*-chroma, and the smaller the h*-value. Thus, a “Y chroma” value has been calculated in this study to link the positive a*-value (red color) and the other b*, C*, and h* values of the colored culture filtrates. The results are presented in Figure 3. In PDB medium with both complex sources of carbon and nitrogen, the four fungal strains belonging to the reddish pigment producers could produce diffusible polyketide red pigments: the culture filtrates exhibited an intense purple, red, or orange-red color, in the darkness, as well as under light conditions (Figure 3A), which is consistent with visual observations of the culture filtrate and the volumetric production of extracellular pigments noticed in these fungal cultures grown in PDB. The highest positive a*-value and positive “Y chroma” value were obtained for the culture filtrate of the marine isolate *Talaromyces* spp. (purple-red color; a*-value up to +63.8) and the strain *P. purpurogenum rubisclerotium* (purple-red color; a*-value up to +51.5). The two other strains, *F. oxysporum* and *T. atroviride*, exhibited lower positive a*-values: up to +35.2 (red color) and +18.7 (orange-red color), respectively.

Furthermore, it is worth noticing that in the “minimal” DMD liquid broth (with only glucose and ammonium sulfate, combined with some metal ions and inorganic salts), only the marine isolate *Talaromyces* spp. and the strain *F. oxysporum* can produce water-soluble reddish pigments: with a corresponding red a*-value from +10 to +20 (Figure 3B). The strain *P. purpurogenum rubisclerotium* grown in this DMD broth produced pinkish pigments exclusively under light conditions, and a pale yellowish pigmentation was noticed for the marine isolate *T. atroviride* grown in DMD. The results confirmed that the same fungal strain did not show the same metabolism of extracellular polyketide pigments in submerged cultures with respect to the medium composition used. Finally, in YCD medium (with glucose, yeast extract, and casamino acids as the complex nitrogen source), only one strain, that is *P. purpurogenum rubisclerotium*, can produce reddish pigments in liquid broth (red a*-value of +42.9 and +43.5 for light and dark culture, respectively) (Figure 3C). Interestingly, the marine isolate *T. atroviride* cultivated in YCD broth produced an orange-red pigmentation exclusively under light conditions, indicating that the production of orange-red pigments is a light-inducible metabolism.

### 3.2. New Extraction Procedure and Nature of the Polyketide Red Pigments Produced by Talaromyces spp. Marine Isolate and P. purpurogenum rubisclerotium Terrestrial Isolate

In order to evaluate the intracellular production and the nature of the red pigments produced by the *Talaromyces* spp. marine isolate and *P. purpurogenum rubisclerotium* terrestrial isolate, a series of six sequential pressurized liquid extractions (PLE) were realized. The sequence of solvents was set to show a decreasing polarity profile, therefore allowing a refined isolation of different pigment molecules, depending on their polarity profiles. The total polyketide secondary metabolite contents extracted from the mycelial biomass, analyzed by measuring the absorbance of all the extracts by spectral analysis at 276 nm and expressed in terms of meqv. of polyketide carmine per g dry cell mass, are shown in Table 1. Thus, the results confirmed that the amount of polyketide pigments produced extra- versus intracellularly varies with different cultivation factors and the choice of fungal strain. The DMD medium favors the production of mycelial biomass, whereas the nutrient composition of the PDB medium favors both the intracellular production of polyketide pigments (up to 69.8 and 116.0 meqv·g^−1^ dry biomass of *Talaromyces* spp. and *P. purpurogenum rubisclerotium*, respectively) and their liberation as extrolites in the liquid broth (up to 278 and 1118 meqv·L^−1^ of culture filtrate of *Talaromyces* spp. and *P. purpurogenum rubisclerotium*, respectively).

**Figure 4:**
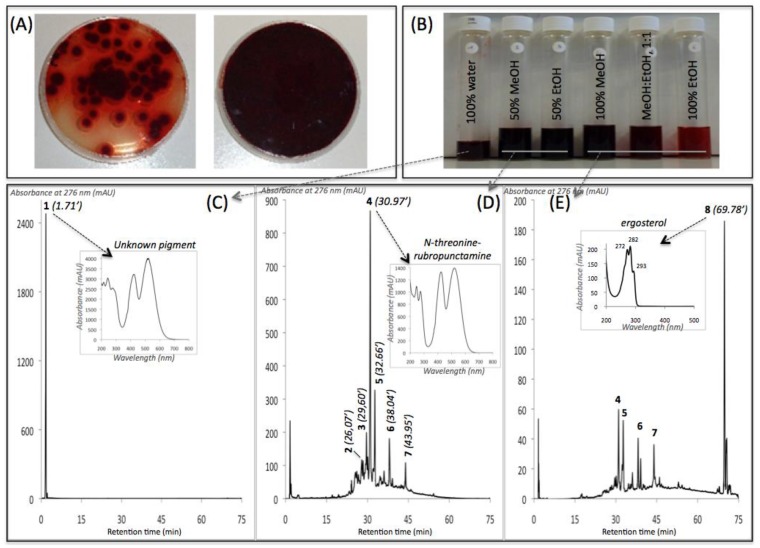
(**A**) Cultures in PDA of the marine isolate *Talaromyces* spp. 305_70; (**B**) liquid extracts obtained after a six-stage pressurized liquid solvent extraction of the mycelium cultivated in PDB submerged culture; (**C**–**E**) chromatograms* of the overall compounds detected by HPLC-DAD in the extracts. *Captions*: PDA (Potato Dextrose Agar); PDB (Potato Dextrose Broth); HPLC-DAD: high-performance liquid chromatogram combined with photo-diode array detection; Rt.: retention time; * the analysed samples were extracted with different solvents. Table 2 reports on all of the identified or assumed compounds detected in all the different samples and, in particular, the 50% aqueous methanolic extract was the most representative, as shown in Figure 4D. Only compound 1 (Rt. 1.71 min), not tentatively identified, was detected in the intracellular aqueous extract (Figure 4C). The main compound 4 (Rt. 30.97 min), identified as *N*-threoninerubropunctamine, was detected mainly in the 50% aqueous methanolic extract (Figure 4D), and compound 8 (Rt. 69.78 min), identified as ergosterol, was detected mainly in 100% methanolic extract (Figure 4E).

The intracellular extracts of the *Talaromyces* spp. marine isolate and *P. purpurogenum rubisclerotium* terrestrial isolate were analyzed by HPLC-DAD. Concerning the polyketide pigments extracted from the mycelial biomass of *Talaromyces* spp. grown in PDB culture, all the intracellular extracts recovered from the six-stage PLE presented an intense purple-red shade (S4). The chromatograms of the intracellular extracts shown in Figure 4 indicated that the intracellular aqueous extract (Figure 4C) was entirely composed of a major colored metabolite (compound **1**; not tentatively identified, >95% *w*/*w* on total compounds in this intracellular aqueous extract) with the retention time (Rt.) of 1.71 min (red polar compound with λ_max_ 215, 244, 276, 418, 514, 524 nm). Then, the 50% aqueous methanolic solution used as a solvent in the six-stage PLE extracted the largest part of other intracellular pigments from *Talaromyces* spp. Six other major colored metabolites were detected in this reddish liquid sample (Figure 4C): compound **2** with Rt. 26.07 min (3.1% *w*/*w* on total secondary metabolites in this intracellular hydroalcoholic extract), compound **3** with Rt. 29.60 min (4.9%), the main compound **4** with Rt. 30.97 min (54.4%), compound **5** with Rt. 32.66 min (14.8%), compound **6** with Rt. 38.04 min (10.9%), and compound **7** with RT 43.95 min (6.3%). In addition, compound **8** with Rt. 69.78 min, identified as ergosterol by UHPLC-HRMS analysis (see below), was essentially detected in the 100% methanolic extract (solvent polarity index of 5.0) (Figure 4E).

**Figure 5:**
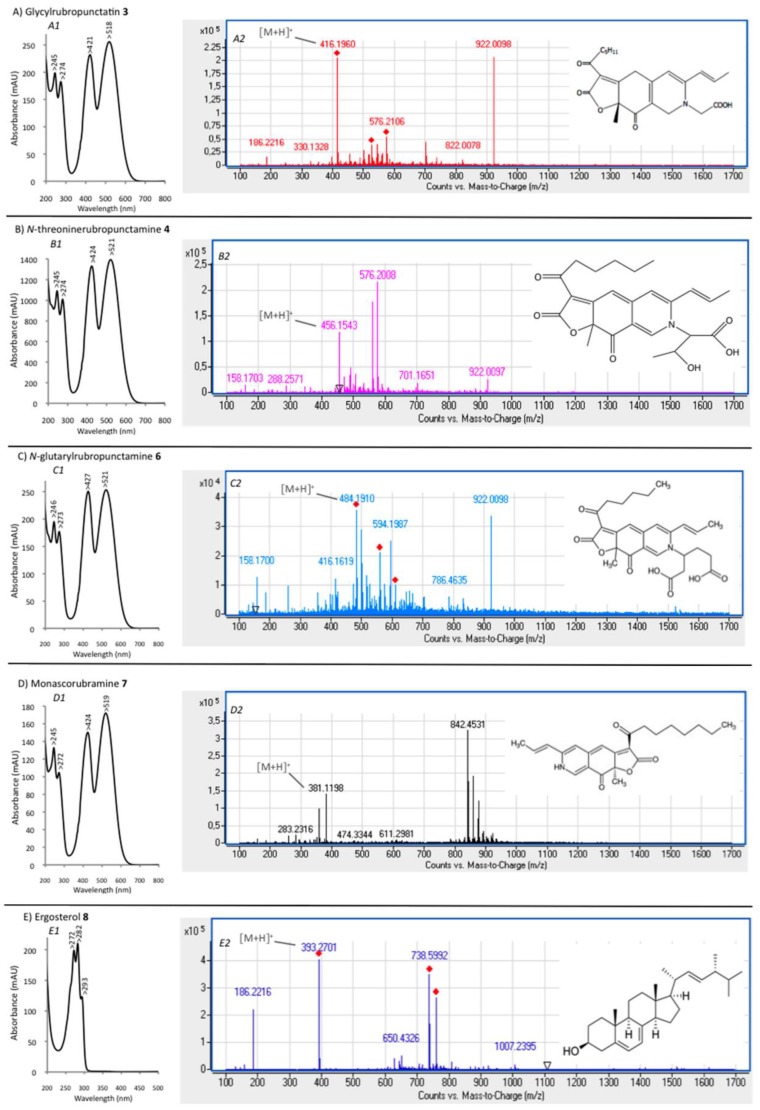
UV-visible absorption (**A1**, **B1**, **C1**, **D1**, **E1**) and mass spectra (**A2**, **B2**, **C2**, **D2**, **E2**) of the identified or assumed compounds detected in intracellular extracts of the marine isolate *Talaromyces* spp. with reference to the chromatograms shown in Figure 4: glycylrubropunctatin **3**, *N*-threoninerubropunctamine **4**, *N*-glutarylrubropunctamine **6**, Monascorubramine **7**, and ergosterol **8**. Compounds **1**, **2** and **5** are red azaphilone-derivative pigments not tentatively identified.

**Figure 6:**
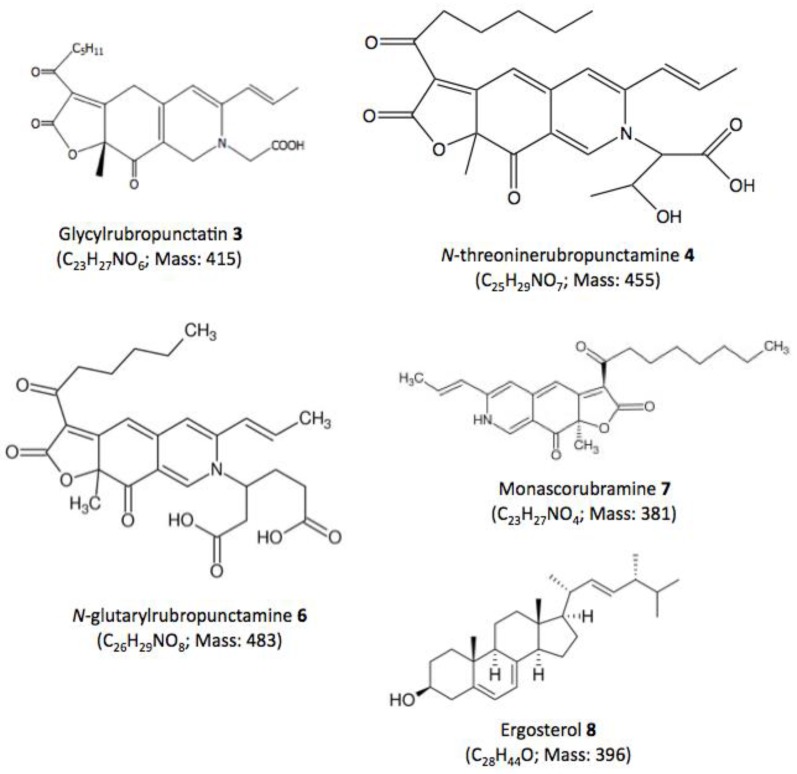
Chemical structures of the identified or assumed compounds detected in the present study in intracellular extracts of the marine isolate *Talaromyces* spp. Formula and calculated nominal masses are shown in parentheses.

The eluted compounds (from compound **1** to **8**) were analyzed by performing UHPLC-HRMS, their absorption spectra were measured from 200–800 nm, and their mass spectra were determined and compared against the spectral library and literature database for identification. The UV-visible absorption and mass spectra of each identified or assumed compound detected in all the different intracellular extracts of the marine isolate *Talaromyces* spp. are shown in Figure 5. The results suggested that the main compound **4** that presents two absorption maxima in the visible region, at λ_max_ at 424 and 521 nm (red color), was identified as the red polyketide azaphilone pigment *N*-threoninerubropunctamine. Indeed, the molecule showed a similar UV-visible absorption spectrum, as well as an ESI-MS molecular ion in positive mode [M + H]^+^ at *m*/*z* 456 as the red pigment *N*-threoninerubropunctamine previously isolated from some species of *Talaromyces* by other authors [25,26]. Presumably, the compound **3** that presents λ_max_ at 421 and 518 nm in the visible region, and the ESI-MS molecular ion observed at *m*/*z* 416 in positive mode [M + H]^+^ seemed to correspond to the red polyketide azaphilone pigment glycylrubropunctatin, which has previously been isolated from *Monascus* cultures [27]. Compound **6** (*N*-glutarylrubropunctamine) and **7** (monascorubramine), which exhibit ESI-MS molecular ions observed at *m*/*z* 484 and *m*/*z* 381 in positive mode [M + H]^+^, respectively, were identified in good agreement with the expected mass of the corresponding molecules previously isolated from some species of *Talaromyces* [25,26]. Finally, the results suggested that compound **8** (with Rt. 69.78 min) seemed to correspond to an ergosterol derivate, according to its absorption spectrum, which was similar to that of an ergosterol molecule, and to the ESI-MS molecular ion observed at *m*/*z* 393 in positive mode [M + H]^+^ relatively close to those of the standard molecule (which in addition to the expected ion *m*/*z* 397, also yielded the same ion at *m*/*z* 393 (major) because ergosterol had undergone desaturation during LC/MS) [28]. This observation is consistent with the findings of previous studies, which have already isolated ergosterol derivates from some fungal species [29,30]. All secondary metabolites detected from intracellular extracts of *Talaromyces* spp. are reported in Table 2, and the chemical structures of all identified or assumed compounds are described in Figure 6.

The three other compounds eluted, i.e., compound **1** (with Rt. 1.71 min; λ_max_ 215, 244, 276, 418, 514, 524 nm; but no signal observed in ESI-MS analysis), compound **2** (with Rt. 26.07 min; λ_max_ 246, 276, 425, 512 nm; ESI-MS molecular ion observed at *m*/*z* 488 in positive mode [M + H]^+^ together with the ion at *m*/*z* 550 [M+Na+CH_3_CN]^+^), and compound **5** (with Rt. 32.66 min; λ_max_ 218, 250, 287, 581 424, 546 nm; ESI-MS molecular ion observed at *m*/*z* 498 in positive mode [M + H]^+^ together with the ion at *m*/*z* 546), presented UV-visible absorption spectra relatively close to those of the *Monascus*-like azaphilone red pigments, which exhibit two absorption maxima in the visible region near 420 and 520 nm (Figure S5). According to this information, these three not tentatively identified metabolites were classified as water-soluble red azaphilone-derivative pigments. Unfortunately, further experiments with a dereplication purpose using UHPLC-HRMS and comparison with a spectral library were not conclusive enough to fully conclude on the nature of these pigmented compounds. Works are in progress in our laboratory to fully characterize the pigments by NMR analysis.

**Figure 7:**
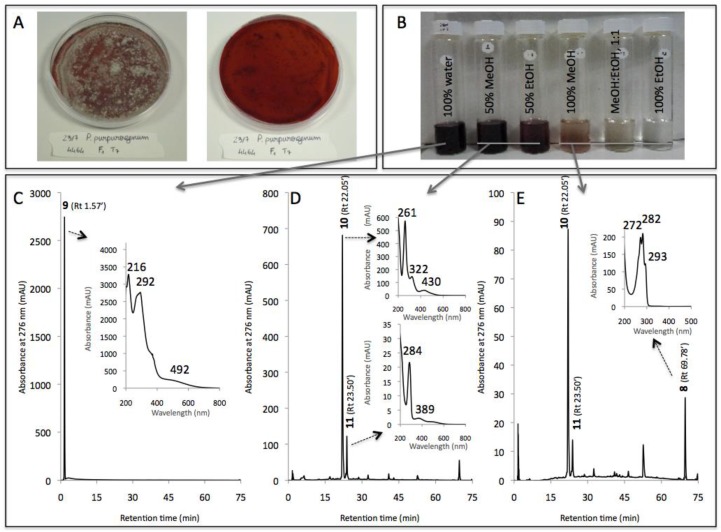
(**A**) Cultures in PDA of the strain *Penicillium purpurogenum rubisclerotium* LCP4464; (**B**) liquid extracts obtained after a six-stage pressurized liquid solvent extraction of the mycelium cultivated in PDB submerged culture; (**C**–**E**) chromatograms * of the overall compounds detected by HPLC-DAD in the different extracts. *Captions*: PDA (Potato Dextrose Agar); PDB (Potato Dextrose Broth); HPLC-DAD: high-performance liquid chromatography combined with photo-diode array detection; Rt.: retention time; *the analysed samples were extracted with different solvents. Table 2 reports the compounds detected in all the different samples and, in particular, the 50% aqueous methanolic extract was the most representative, as shown in Figure 7D. Only compound **9** (Rt. 1.57 min; not tentatively identified) was detected in the aqueous extract (Figure 7C), the main compound **10** (Rt. 22.05 min; not tentatively identified) was detected mainly in the 50% aqueous methanolic extract (Figure 7D), and ergosterol 8 (Rt. 69.78 min) was detected mainly in 100% MeOH extract (Figure 7E).

For the purpose of comparison, Table 2 summarized the compounds eluted in all the different intracellular extracts of the strain *P. purpurogenum rubisclerotium*. Regarding the intracellular pigments extracted from this fungal mycelium cultivated in PDB, the chromatograms shown in Figure 7 indicated that the aqueous extract was entirely composed of a major secondary metabolite with Rt. 1.57 min (compound **9** with the following λ_max_: 216, 292, 492 nm; not tentatively identified), which is different from compound **1** detected from the aqueous extract of *Talaromyces* spp. Similarly, the 50% aqueous methanolic solution used as a solvent in the six-step PLE extracted the largest part of other intracellular pigments from this *P. purpurogenum rubisclerotium* strain: two other major colored metabolites were detected in this reddish liquid extract: the main compound **10** with Rt. 22.05 min that presents λ_max_ at 262, 322, 430 nm (not tentatively identified); and compound **11** with Rt. 23.50 min exhibited λ_max_ at 284 and 389 nm (not tentatively identified). Further analytical experiments and comparisons with the spectral library were not conclusive enough to fully conclude on the nature of these pigmented compounds from intracellular extracts of *P. purpurogenum rubisclerotium*. The last compound eluted with Rt. 69.78 min was essentially detected in the 100% methanolic extract, and seemed to correspond to the same ergosterol **8** derivate previously isolated from intracellular extracts of *Talaromyces* spp. marine isolate.

## 4. Discussion

The 11 fungal strains from the terrestrial environment investigated in this study were firstly selected according to the capacity of analogous fungal species cited in the literature to produce pigments in solid and/or submerged cultures. The four local marine isolates were selected according to previous works performed in our laboratory (data not published) based on their extracellular pigment producing ability in submerged cultures. We identified four strains, *P. purpurogenum rubisclerotium*, *F. oxysporum*, and two marine isolates sampled from the lagoon on the west coast of La Reunion Island identified as *Talaromyces* spp. and *Trichoderma atroviride*, as potential pigment producers that produced polyketide red pigments. Regarding the pigment yield, there are generally two natural ways in which the concentration of pigments can be increased: either by improving the fungal growth, or by increasing the intracellular accumulation of pigments. The problem, however, lies with the difficulty in increasing both the biomass and the pigment production, which would be optimal for industrial production. Biomass and pigment yields tend to be negatively correlated. An increase in the biomass yield is connected to the abundance of nutrients in the medium, whereas pigment production tends to be increased under nutrient-poor conditions and/or external stresses, as a protective mechanism [31]. Therefore, the relationship between biomass and pigment production needs to be fully elucidated in order to optimize and control pigment production in submerged fungal culture.

Our results confirmed that same fungal strain did not show the same metabolism of extracellular pigments in submerged cultures (due to the excretion of diffusible pigments in liquid broth) with respect to the medium composition used. For example, the highest water-soluble extrolites production was obtained for the culture filtrate of the strain *P. purpurogenum rubisclerotium* in PDB liquid broth, although its biomass concentration was very low in this medium. As a result, the nutrient composition of the PBD liquid broth allows one to associate the high production and excretion of extracellular water soluble pigments with the low formation of mycelial biomass. Along similar lines, two other strains belonging to the reddish pigment producers that are *F. oxysporum* and *T. atroviride* presented the highest extracellular polyketide pigment productivities in PDB submerged cultures, but the lowest contents of mycelial biomass. These results confirmed the difficulty in increasing both the mycelial biomass and the extracellular pigment production at the same time, which would be optimal for industrial production. Surprisingly, the marine isolate *Talaromyces* spp. presented a different behavior towards the medium nutrient composition. It simultaneously showed a relatively high mycelial biomass content (above 5.5 g·L^−1^ of dry biomass) and a strong content in extracellular polyketide pigments in culture filtrate, using submerged fermentation in PDB.

In general, the secretion of extracellular pigments is favored over intracellular production at an industrial scale, as it implies less downstream extraction processes. Furthermore, there are several submerged fermentation techniques that can be used, such as fed-batch or continuous mode approaches, in order to achieve optimal pigment yields [31]. Our results suggested that an increase in the mycelial biomass yield is mainly connected to the abundance of simple source of carbon and nitrogen in the medium (f.i. in DMD broth), whereas the production and especially secretion of extracellular polyketide pigments seemed to be increased with an abundance of complex sources of carbon (e.g., starch from PDB) and nitrogen (e.g., amino acids and proteins from PDB) combined with some essential cofactors like calcium, magnesium, phosphor, iron, manganese, zinc, and copper (all present in PDB broth). Indeed, it has been previously reported in the literature that the use of a complex nitrogen source or the addition of individual amino acids tends to influence the number, type, and excretion rates of different pigments that are formed in submerged fungal cultures [31]. It has been suggested that the stimulating effect of amino acids on the production or excretion of pigments is caused by an increase in solubility due to the greater hydrosolubility profile of the pigments when bound to an amino acid than the native pigment on its own. It has also been reported that fungal pigments access the aqueous environment by association with proteins or other polar compounds [31].

In this study, the volumetric production of polyketide extrolites secreted by each strain in the culture filtrate was estimated by a spectrophotometer on the basis of the measured absorbance at 276 nm, and expressed as milli-equivalents of polyketide carmine per liter of liquid broth. This estimation is a value proportional to the total polyketide pigment concentration in the culture filtrate. Indeed, most polyketide-derived pigments are characterized by absorption bands in the UV domain (near 240–260 nm) due to the benzene structure and most of them also presented one UV absorption peak near 276 nm, while the maximum absorbencies in the visible region of these polyketide-derived pigments varied from the range of 400 (yellow pigments) to 500 nm (red pigments). Examples include the anthraquinone red pigment carmine which exhibited a λ_max_ at 276 nm, the naphtoquinone red pigment bikaverin (λ_max_ at 253 and 276 nm) isolated from *Fusarium* species, and most *Monascus*-like pigments with a common azaphilone skeleton isolated from *Penicillium* and *Talaromyces* species such as yellow ankaflavin and red monascorubramine (λ_max_ at 276 nm). Furthermore, our data indicate that the positive a*-value, i.e., the red colorimetric coordinate in the CIE L*a*b* color system, and the measured absorbance at 276 nm of the pigmented culture filtrates, tend to be slightly positively correlated for some fungal strains belonging to the reddish pigment producers (data not shown), which justifies the use of this wavelength as the detection value for all polyketide pigments produced by the fungal strains.

For the quantification and characterization of intracellular polyketide pigments, a greener protocol for the pigment extraction, that is a pressurized liquid extraction (PLE) procedure using environmental-friendly solvents, has been investigated. To our knowledge, there is no standard method for polyketide-derived pigment extraction from mycelial biomass. However, based on literature works, the commonly used process involves mixing dried biomass with an organic solvent (like ethyl acetate, chloroform, etc.) or a mixture, followed by the mechanical disruption of the cells, and subsequent centrifugation or filtration (solid-liquid extraction) [6,31]. In general, cell disruption is most necessary for the higher recovery of intracellular fungal pigments. Many different methods for cell disruption have been suggested by authors, including mechanical (sonification) and non-mechanical disruptions (chemical extraction processes) [6,31]. The preferred extraction procedure is based on a quick process that efficiently releases all of the intracellular pigments from the matrix into the solution without altering them, while using environmental-friendly solvents, if possible [6,31]. Moreover, the polarity of the biocompounds to be extracted is the determinant in the selection of the extraction solvent [32]. Therefore, and as the fungal biomass is likely to contain a mixture of pigments of various natures, multi-stage extraction methods using solvents of a different polarity are required, in order to obtain the most exhaustive pigment composition profile for each fungal strain tested.

In our study, lyophilized mycelial biomass was subjected to a six-stage PLE using environmental-friendly solvents including water, methanol, and ethanol, all already allowed and widely used in the EU and in the US for the extraction of natural food colorants. The protocol was illustrated in the intracellular pigment extracts of *P. purpurogenum rubisclerotium* and *Talaromyces* spp. analysed by HPLC-DAD and UHPLC-HRMS. The PLE system offers the advantages of considerably reducing the extraction times and solvent amounts to be used, as well as using more sustainable solvents such as water, ethanol, and methanol with higher extraction efficiencies [32]. It consists of a solid-liquid extraction process carried out at a high temperature (50–100 °C) and elevated pressure (10–150 MPa) in order to maintain the solvent(s) at a liquid state when applied to the sample [18,19]. The high pressures help in forcing the solvent into the cell pores, while the increased temperature enhances the extraction kinetics. Additionally, the use of increased pressure during the extraction protects the compounds of interest from oxygen, and ensures a higher quality of the recovered biochemical. Then, PLE is considered as a promising extraction process particularly appropriate for polar compounds. Moreover, being able to use common and non-toxic solvents is of great interest when dealing with compounds, which are intended to be used in food and cosmetic products, from health and safety, environmental, and economic prospects, as it would result in less downstream purification processes, less waste treatments, and less energy and solvent expenses. Furthermore, solvents such as ethanol and methanol can be produced from carbon neutral homoacetogenic gas fermentation and biogas produced from waste, respectively, strengthening the sustainability of such process. The six-stage PLE procedure gives encouraging results in terms of the efficiency and selectivity of the pigments extracted from the fungal mycelia. It also paves the way for further optimizations of the solvent mixture which can be used to isolate specific pigments. In the meantime, extraction results confirmed that the minimal ‘DMD’ medium favors the production of mycelial biomass to the detriment of pigment production. On the other hand, the nutrient composition of the PDB medium favors both the intracellular production of pigments (up to 69.8 and 116.0 meqv·g^−1^ dry biomass of *Talaromyces* spp. and *P. purpurogenum rubisclerotium*, respectively) and their liberation as extrolites in the liquid broth (up to 277 and 1178 meqv·L^−1^ culture filtrate in submerged culture of *Talaromyces* spp. and *P. purpurogenum rubisclerotium*, respectively). Thus, an efficient and sustainable multi-step extraction process has been developed, allowing a sequential extraction of a wide panel of pigmented molecules based on their respective polarity. Such a process could be further applied to other types of sample matrices for biochemical content determination.

The 50% aqueous methanolic solution used as extraction solvent in the six-stage PLE procedure recovered the largest part of intracellular pigments from the two fungal mycelia. Seven major colored metabolites were detected in intracellular pigment extracts of *Talaromyces* spp. marine isolate: all corresponding to *Monascus*-like pigments with a common azaphilone skeleton, including the main water-soluble azaphilone red pigment identified as *N*-threoninerubropunctamine **4** (non-toxic compound), followed by three other assumed *Monascus*-like azaphilone pigments such as *N*-glutarylrubropunctamine **6**, monascorubramine **7**, and glycylrubropunctatin **3**. The generally recognized pathway proposes that the orange *Monascus* and *Monascus*-like azaphilone pigments, including monascorubrin and rubropunctatin (insoluble in water), are formed by the esterification of a beta-ketoacid (from the fatty acid synthase pathway) to the chromophore (derived from the polyketide synthase pathway). Then, the reduction of the orange *Monascus* and *Monascus*-like azaphilone pigments yields the yellow *Monascus*-like azaphilone pigments (monascin, ankaflavin). In contrast, amination of the orange pigments with amino group-containing compounds in the medium (proteins, amino acids and nucleic acids) leads to the water-soluble red *Monascus* and *Monascus*-like azaphilone pigments, including the monascorubramine **7** and rubropunctamine derivates like *N*-threoninerubropunctamine **4** and *N*-glutarylrubropunctamine **6** identified in this study [33].

For the purpose of comparison, three other red colored metabolites (not tentatively identified in this study) were detected in intracellular extracts of *P. purpurogenum rubisclerotium* terrestrial isolate (also known under its *Talaromyces pinophilus* name, according to Frisvad et al. [14]). Ergosterol **8** was also isolated as a minor compound in the intracellular pigment extracts from the two fungal mycelia.

## 5. Conclusions

We report on this article newly isolated polyketide red pigment producing ascomycetous fungi namely *Penicillium purpurogenum rubisclerotium*, *Fusarium oxysporum*, and two fungal isolates of marine origin identified as *Talaromyces* spp. and *Trichoderma atroviride*, sampled from the Reunion Island marine habitat. A new sustainable pigment extraction method was used, that is a six-stage pressurized liquid extraction protocol, for advanced mycelial pigment extraction. Such methods, which use less and non-toxic solvents, have not been used on fungal matrixes thus far. The more promising strain was the non-mycotoxigenic fungus *Talaromyces* spp. of marine origin. According to its pigment production capacity and the emerging natural red pigments in the global market, this fungal strain represents an interesting example of a microorganism which can produce a variety of interesting bioactive compounds like natural red colorants. In addition, the production of secondary metabolites is important for the chemotaxonomic characterization of this fungal isolate. The main pigment produced by this strain has been characterized as *N*-threoninerubropunctamine, a non-toxic red *Monascus*-like azaphilone pigment. Knowledge of the intracellular and extracellular pigment productions by this fungus in submerged culture is important for the development of a high-performance industrial fermentation process for polyketide red pigment production. Further studies are necessary for future applications in the dye industry.

## Figures and Tables

**Table 1:** Biomass and extracellular versus intracellular pigment production by the marine isolate *Talaromyces* spp. and terrestrial isolate *P. purpurogenum rubisclerotium* in submerged cultures.

Fungal Strain	Broth	Biomass	Extracellular Pigments	*a*-Value (CIELab)	Intracellular Pigments Extracted	
		(g/L)	(meqv. Carmine/L)	(Red Color)	(meqv/g Biomass) or (meqv/L Culture)	
30570	in DMD	**6.2–7.1**	18–31	10.8–13.4	23.8–24.7	148–176
	in PDB	5.0–5.5	**208–278**	**63.8–65.8**	**49.4–69.8**	**247–384**
	in YCD	1.6–2.0	-	5.3–5.6	44.3–48.5	71–97
4464	in DMD	**8.4–8.5**	1–189	0.7 – 0.9	57.9–83.2	**487–707**
	in PDB	1.0–1.3	**685–1118**	**49.9–51.5**	**69.2–116.0**	90–116
	in YCD	1.6–1.7	666–680	42.8–43.5	22.5–71.2	36–121

30570: marine isolate of *Talaromyces* spp.; 4464: terrestrial isolate of *Penicillium purpurogenum rubisclerotium*; DMD: Defined Minimal Medium broth; PDB: Potato Dextrose Broth. YCD: Yeast Casamino acids Dextrose Broth; Values corresponding to the main productivities are noticed in bold.

**Table 2:** Overall compounds detected by HPLC-DAD and UHPLC-HRMS in intracellular extracts (IE) of the marine isolate of *Talaromyces* spp. and terrestrial isolate of *P. purpurogenum rubisclerotium* cultivated in PDB submerged culture, with reference to the chromatograms shown in Figure 4 and Figure 7.

Compound No. R.t. (mn)		λmax (nm)	Solvent	Tentative Identification	Mol. Peak (*m*/*z*)
IE of *Talaromyces* spp. (305_70)					
1	1.71	215, 244, 276, 418, 514, 524	water	n.i.	n.d.
2	26.07	246, 276, 425, 512	MeOH	n.i.	488 [M + H]^+^
3	29.60	245, 274, 421, 518	MeOH	Glycylrubropunctatin	416 [M + H]^+^
4	**30.97**	**245, 274, 424, 521**	**MeOH**	***N*-threoninerubropunctamine**	**456** [M + H]^+^
5	32.66	218, 250, 287, 424, 546	MeOH	n.i.	498 [M + H]^+^
6	38.04	246, 273, 427, 521	MeOH	*N*-glutarylrubropunctamine	484 [M + H]^+^
7	43.95	245, 272, 424, 519	MeOH	Monascorubramine	381 [M + H]^+^
8	69.78	272, 282, 293	MeOH	Ergosterol	393 [M + H]^+^
IE of *Penicillium purpurogenum rubisclerotium* (4464)					
8	69.78	272, 282, 293	MeOH	Ergosterol	393 [M + H]^+^
9	1.57	216, 292, 492	MeOH	n.i.	n.d.
10	**22.05**	**262, 322, 430**	**MeOH**	n.i.	n.d.
11	23.50	284, 389	MeOH	n.i.	n.d.

IE: Intracellular extract; PDB: Potato Dextrose Broth; Main compound in bold; n.i. = not tentatively identified.

## Supplementary Materials

The following are available online at www.mdpi.com/2309-608X/3/3/34/s1.
