# Supplementary material for: Production and New Extraction Method of Polyketide Red Pigments Produced by Ascomycetous Fungi from Terrestrial and Marine Habitats
Source: J Fungi (Basel). 2017 Jun 28;3(3):34. doi: 10.3390/jof3030034 (PMC5715940; doi:10.3390/jof3030034)

Article

Production and new extraction method of polyketide red pigments produced by ascomycetous fungi from terrestrial and marine habitats

Juliana Lebeau [1], Mekala Venkatachalam [1], Mireille Fouillaud [1], Thomas Petit [2], Francesco Vinale [3], Laurent Dufossé [1] and Yanis Caro [1,*]

1 Laboratoire de Chimie des Substances Naturelles et des Sciences des Aliments (LCSNSA), Université de la Réunion, F-97490 Sainte-Clotilde, Ile de la Réunion, France; juliana.lebeau@gmail.com (J.L.); mekalavenkat@gmail.com (M.V.); mireille.fouillaud@univ-reunion.fr (M.F.); laurent.dufosse@univ-reunion.fr (L.D.)

2 UMR QualiSud, Université de la Réunion, IUT, F-97410 Saint-Pierre, Ile de la Réunion, France; thomas.petit@univ-reunion.fr

3 Istituto per la Protezione Sostenibile delle Piante (IPSP-CNR) and Dipartimento di Agraria, Università degli Studi di Napoli Federico II, 80055 Portici, NA, Italy; francesco.vinale@ipsp.cnr.it

* Correspondence: yanis.caro@univ-reunion.fr; Tel.: +262-262-483-361

Supplementary materials

Figure S1: Absorption spectrum and calibration curve (concentration vs. absorbance) of carmine standard solution in deionized water measured at λ_{max} 276 nm (that has been found by first scanning the sample for λ_{max})

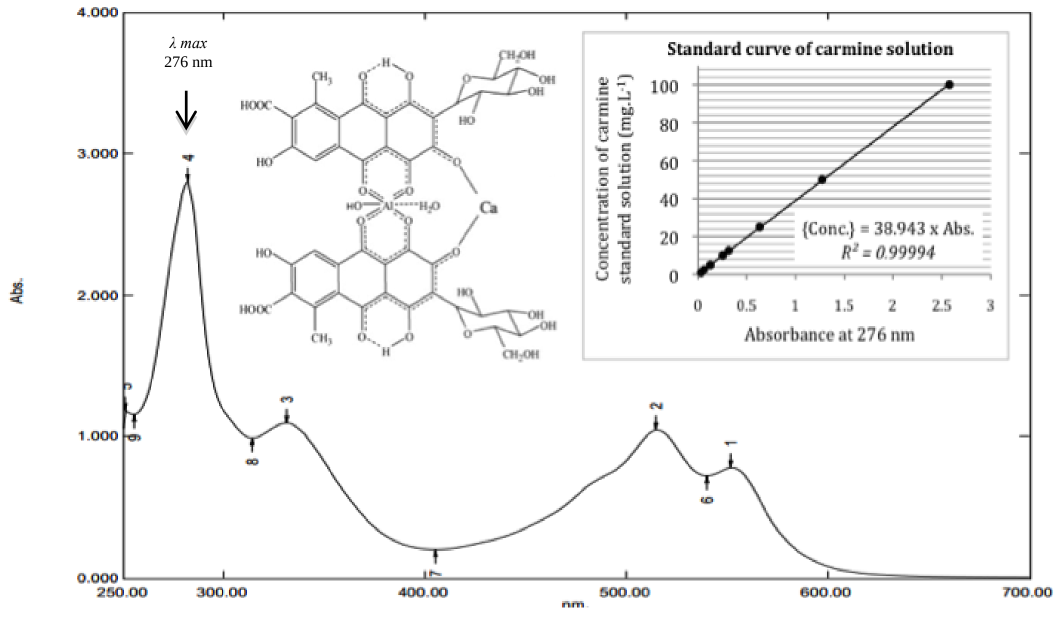

Figure S2: Schematic representation of the PLE (pressurized liquid extraction) protocol using a six-stage solvent extraction method with water, methanol and ethanol.

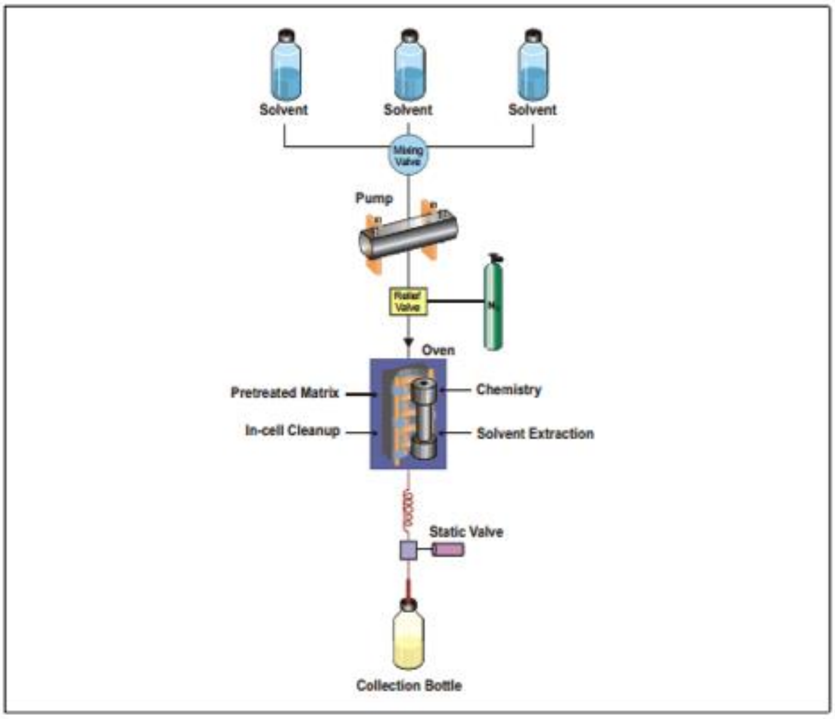

Six-stage solvent extraction	Polarity index*
Solvent n°1 - water	10.0
Solvent n°2 – 50% aqueous methanol	7.5
Solvent n°3 – 50% aqueous ethanol	7.0
Solvent n°4 - methanol	5.0
Solvent n°5 – methanol/ethanol (50:50, v/v)	4.5
Solvent n°6 - ethanol	4.0

Figure S3: Pictures and shades of the submerged cultures of the fungal strains after 7 days of fermentation.

Legends: PDB (Potato Dextrose Broth), YCD (Yeast Casamino Dextrose broth), DMD (Defined Minimal Dextrose broth), MH: strains isolated from Reunion Island marine habitats; TE: strains from terrestrial environments; For composition of the media, see section '*Materials & Methods*'; *Strains collected from marine biotopes of La Reunion island's reef flat.

<table>
<tr><th rowspan="2">Strains</th><th colspan="2">PDB medium</th><th colspan="2">YCD medium</th><th colspan="2">DMD medium</th></tr>
<tr><th>culture</th><th>shade</th><th>culture</th><th>shade</th><th>culture</th><th>shade</th></tr>
<tr><td>LCP4464 (TE)
❶ Penicillium purpurogenum rubisclerotium</td><td></td><td>purple</td><td></td><td>purple</td><td></td><td>pink-red</td></tr>
<tr><td>305_70 (MH)
❷ Talaromyces sp</td><td></td><td>purple</td><td></td><td>pink-red</td><td></td><td>dark-pink</td></tr>
<tr><td>LCP531 (TE)
❸ Fusarium oxysporum</td><td></td><td>red</td><td></td><td>pinkish</td><td></td><td>red</td></tr>
<tr><td>305_55 (MH)
❹ Trichoderma atroviride</td><td></td><td>orange-red</td><td></td><td>orange-red (only in light)</td><td></td><td>pale yellow</td></tr>
<tr><td>LCP4890 (TE)
Penicillium purpurogenum</td><td></td><td>pinkish</td><td></td><td>pinkish</td><td></td><td>white-pinkish</td></tr>
<tr><td>LCP2226 (TE)
Dreschlera cynodontis</td><td></td><td>brown</td><td></td><td>brown</td><td></td><td>black</td></tr>
<tr><td>LCP3684 (TE)
Penicillium erythromellis</td><td></td><td rowspan="8">yellow</td><td></td><td rowspan="3">yellow</td><td></td><td rowspan="4">creamy white</td></tr>
<tr><td>T22 (TE)
Trichoderma harzianum</td><td></td><td></td><td></td></tr>
<tr><td>LCP4158 (TE)
Penicillium oxalicum</td><td></td><td></td><td></td></tr>
<tr><td>LCP5511 (TE)
Aspergillus repens</td><td></td><td></td><td>orange</td><td></td></tr>
<tr><td>PA9 (MH)
Talaromyces verruculosus</td><td></td><td></td><td rowspan="5">yellow</td><td></td><td>Pinkish (only in darkness)</td></tr>
<tr><td>LCP3404 (TE)
Trichoderma harzianum</td><td></td><td></td><td></td><td>pale yellow</td></tr>
<tr><td>LCP3391 (TE)
Paecilomyces farinosus</td><td></td><td></td><td></td><td>Pale green</td></tr>
<tr><td>B34 (MH)
Aspergillus sydowii (B)</td><td></td><td></td><td></td><td>white-pinkish</td></tr>
<tr><td>LCP3531 (TE)
Trichoderma polysporum</td><td></td><td>dark green</td><td></td><td></td><td>light green</td></tr>
</table>

Figure S4: Color of the liquid samples obtained after 6-stage pressurized liquid solvent extraction (PLE) of pigments from mycelium of *Penicillium purpurogenum rubisclerotium* (LCP4464) and marine isolate of *Talaromyces sp* (305_70) cultivated in submerged culture after 7 days of fermentation.

Legends: PDB (Potato Dextrose Broth), DMD (Defined Minimal Dextrose broth), YCD (Yeast Casamino Dextrose broth)

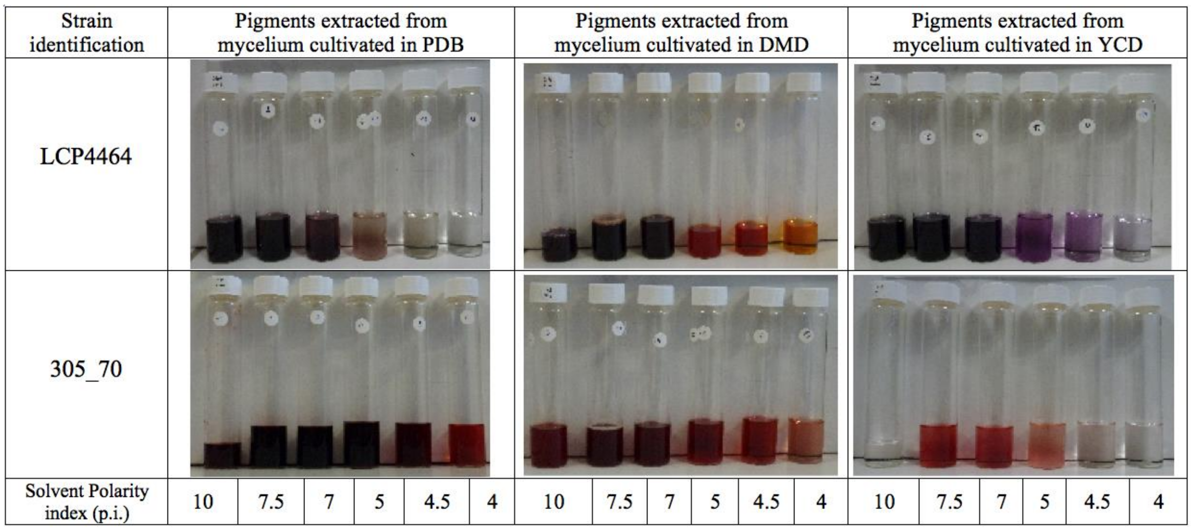

Figure S5: UV-visible absorption and mass spectra of the not tentatively identified compound **2** and compound **5** from intracellular extracts of *Talaromyces spp.*

Compound no 02 : Not tentatively identified in this study

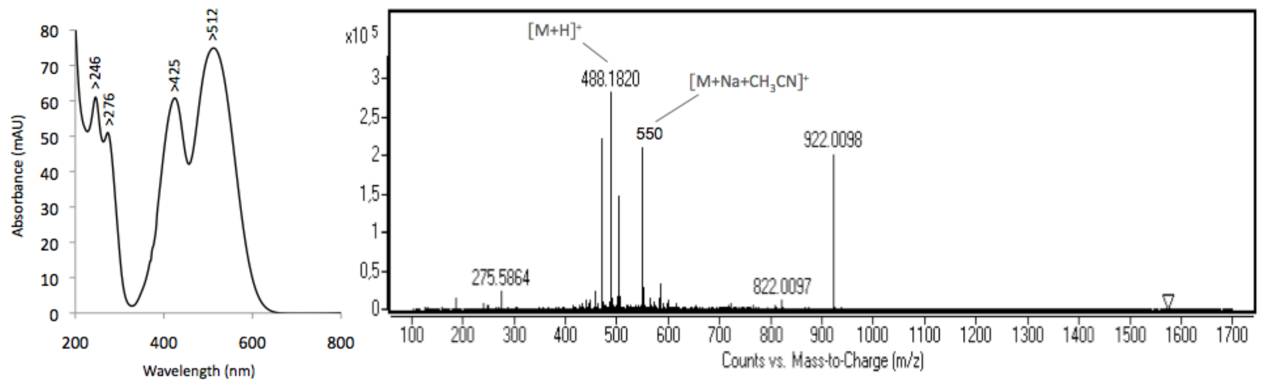

Compound no 05 : Not tentatively identified in this study

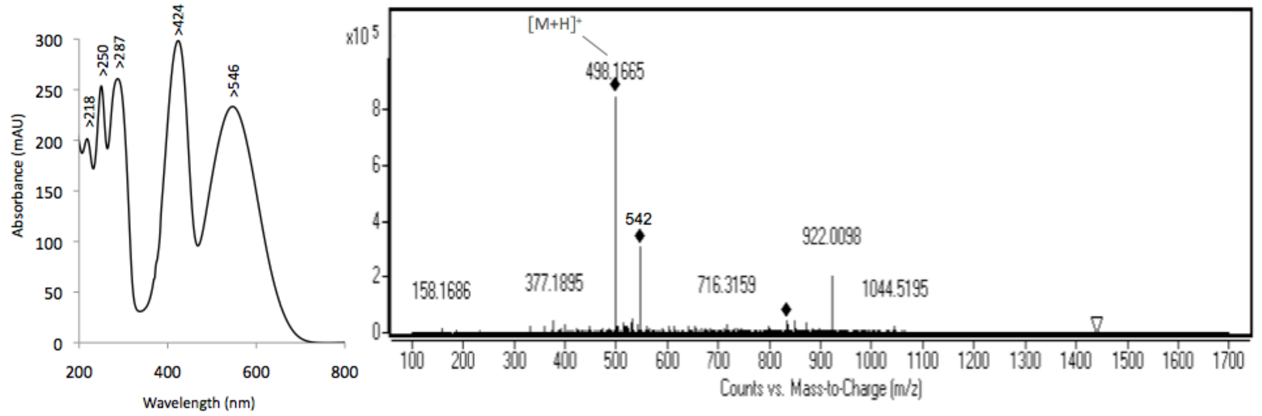

Supplement: Supplementary file 1 [file jof-03-00034-s001.pdf]
